# The diaphragmatic electrical activity during spontaneous breathing trial in patients with mechanical ventilation: physiological description and potential clinical utility

**DOI:** 10.1186/s12890-024-03077-8

**Published:** 2024-05-30

**Authors:** Shitong Diao, Shan Li, Run Dong, Wei Jiang, Chunyao Wang, Yan Chen, Jingyi Wang, Shuhua He, Yifan Wang, Bin Du, Li Weng

**Affiliations:** Medical Intensive Care Unit, State Key Laboratory of Complex Severe and Rare Diseases, Peking Union Medical College, Peking Union Medical College Hospital, Chinese Academy of Medical Sciences, Beijing, China

**Keywords:** Diaphragm, Electrical activity of the diaphragm (EAdi), Mechanical ventilation, Respiratory drive, Ventilator weaning

## Abstract

**Backgrounds:**

Increased respiratory drive has been demonstrated to correlate with weaning failure, which could be quantified by electrical activity of the diaphragm (EAdi). We described the physiological process of EAdi-based parameters during the spontaneous breathing trial (SBT) and evaluated the change of EAdi-based parameters as potential predictors of weaning failure.

**Methods:**

We conducted a prospective study in 35 mechanically ventilated patients who underwent a 2-hour SBT. EAdi and ventilatory parameters were continuously measured during the SBT. Diaphragm ultrasound was performed before the SBT and at the 30 min of the SBT. Three EAdi-based parameters were calculated: neuro-ventilatory efficiency, neuro-excursion efficiency and neuro-discharge per min.

**Results:**

Of the thirty 35 patients studied, 25 patients were defined as SBT success, including 22 patients weaning successfully and 3 patients reintubated. Before the SBT, neuro-excursion efficiency differed significantly between two groups and had the highest predictive value for SBT failure (AUROC 0.875, *p* < 0.01). Early increases in EAdi were observed in SBT, which are more prominent in SBT failure group. One minute, changes in EAdi and neuro-discharge per min also predicted weaning outcome (AUROCs 0.944 and 0.918, respectively).

**Conclusions:**

EAdi-based parameters, especially neuro-excursion efficiency and changes in neuro-discharge per min, may detect impending weaning failure earlier than conventional indices. EAdi monitoring provides physiological insights and a more tailored approach to facilitate successful weaning. Further research should validate these findings and explore the utility of combined EAdi and diaphragm ultrasound assessment in weaning ICU patients from mechanical ventilation.

**Trial Registration:**

Registered at ClinicalTrials.gov on 20 September 2022 (Identifier: NCT05632822).

**Supplementary Information:**

The online version contains supplementary material available at 10.1186/s12890-024-03077-8.

## Background

In intensive care units (ICUs), timely weaning from mechanical ventilation is crucial for patients recovering from respiratory failure. Delayed or premature liberation from mechanical ventilation has been associated with increased mortality rates and prolonged ICU stays [1, 2]. The weaning processes, particularly spontaneous breathing trials (SBTs) in the ICU are typically guided by clinical signs and experience [3, 4]. Despite adequate evaluation, 20–30% of patients experience unexpected mechanical ventilation liberation failure [5].

Over the years, several parameters have been developed to assess weaning readiness from mechanical ventilation and predict weaning failure, however, their efficacy were unsatisfactory [6]. As the most commonly reported parameter, the rapid shallow breathing index (RSBI) is calculated as the ratio of respiratory rate (RR) to tidal volume (VT) [7]. An RSBI value exceeding 105 min/L is indicative of breathing exhaustion. Nonetheless, RSBI has shown only moderate sensitivity and poor specificity in clinical practice [7, 8]. Diaphragm ultrasound is a non-invasive and feasible method for evaluating respiratory muscle function and the cause of weaning failure [9]. However, its limited a sensitivity and positive predictive value hinder its effectiveness as monitoring indicator during SBTs [10, 11]. Given that the success of weaning is contingent upon the equilibrium among respiratory system load, respiratory muscle strength, and respiratory drive, there remains an ongoing requirement for a reliable, accurate, and pragmatic approach to forecast weaning outcomes [12, 13].

Patients experiencing weaning failure might have respiratory load or weak respiratory muscles, leading to an increase in neural respiratory drive as a compensatory mechanism [13]. Therefore, elevated neural drive during the SBT might be an interesting surrogate predictor for weaning failure. The electrical activity of the diaphragm (EAdi) had been utilized to reflect and quantify neural respiratory drive [14, 15]. Previous studies suggested that changes in EAdi could predict SBT failure earlier than conventional parameters [16, 17]. However, the precise usefulness of early changes in EAdi parameters during SBTs is still uncertain.

This study aimed to describe the physiological behavior of SBT by EAdi-based parameters and evaluate the predictive value of EAdi-based parameters for weaning failure. We evaluated neuro-ventilatory efficiency [17, 18], a surrogate for the ability to convert nerve drive into inspiratory tidal volume; neuro-excursion efficiency, a surrogate for the conversion of nerve impulses into muscle movements; and neuro-discharge per min, a surrogate for the total neural drive discharge per unit time.

## Materials and methods

The prospective observational study was performed in a 15-bed medical intensive care unit (ICU) at the Peking Union Medical College Hospital, which is a tertiary teaching center located in Beijing, China, from September 2022 to March 2023. The original study protocol was approved by the institutional review board of Peking Union Medical College Hospital (K2603). The trial was registered at ClinicalTrials.gov (NCT05632822), and informed consent was obtained. Patients were not included in the design, or conduct, or reporting, or dissemination plans of our research.

### Patients

In the study, all adult patients who underwent invasive mechanical ventilation for over 48 h due to acute respiratory failure were eligible for enrollment if they were considered ready for weaning trials. Patients who had phrenic nerve injury, unstable respiratory rhythm, or contraindications for gastric tube, such as esophagogastric varices, upper gastrointestinal perforations, or esophageal surgery were excluded. After admission, the participants were ventilated with the Servo-U ventilator (Maquet, Solna, Sweden), and experienced doctors inserted an EAdi catheter (Maquet, Solna, Sweden) as per the manufacturer’s recommendations before the start of SBT [19]. Details for positioning the EAdi catheter and checking positions were shown in the Supplement Table S1.

### Study protocol

All patients underwent daily screening to determine their readiness for SBT. SBT were conducted according to the recommendations from the International Consensus Conference on weaning [20]: (1) resolving the primary cause of mechanical ventilation; (2) arterial partial pressure of oxygen (PaO_2_)/ fraction of inspired oxygen (FiO_2_) ≥ 200 and positive end-expiratory pressure (PEEP) 4–5 cmH_2_O; (3) Pressure support (PS) level with 10 cmH_2_O for at least 24 h with stable respiration; (4) arterial pH>7.32; (5) rapid shallow breathing index (RSBI)<105 (min·L)^−1^ and respiratory rate (RR)<30 breaths/min; (6) stable hemodynamics (heart rate ≤ 110 bpm, mean artery pressure ≥ 65 mmHg, norepinephrine ≤ 0.1 µg/kg/min). When initiating the SBT, the PS was reduced from 10 cmH_2_O to 0 cmH_2_O, while the PEEP was maintained at 4-5cmH_2_O. The SBT was to be performed for 120 min and patients were reconnected to mechanical ventilation if any of the following events occurred: (1) breathing frequency > 35 breaths/min or < 6 breaths/min; (2) blood oxygen saturation (SpO_2_)<90% with unchanged FiO_2_; (3) new cardiac arrhythmia, heart rate or mean artery pressure increasing>10%; (4) agitation, diaphoresis or complaints of dyspnea [5]. After evaluating the patients’ airway protection,, airway secretions, cough strength, and level of consciousness, two experienced ICU doctors determined whether to extubate patients who had successfully completed the SBT and reconnected to mechanical ventilation for at least 1 h after the SBT [21]. Weaning failure was defined as SBT failure, reintubation, or the use of noninvasive ventilation within 48 h of extubation due to labored breathing.

### Data acquisition and analysis

During the initial 30 min of SBT, we collected EAdi-based parameters and ventilatory parameters at 6 time points, including 0 min (PS 10cmH_2_O) and 1, 5, 10, 20, and 30 min during the SBT. We obtained three measurements at each point and averaged all parameters according to previous studies [22, 23]. EAdi waveforms and other ventilatory parameters were sampled at 100 Hz through an RS232 cable connected to a laptop with dedicated software (Servo-tracker software version 4.2, Solna, Sweden).

We calculated EAdi as EAdi peak minus EAdi minimum. The area under the curve of EAdi from baseline to peak was represented as EAdi_AUC_. Neuro-ventilatory efficiency was calculated as VT divided by EAdi. Neuro-discharge per min was calculated as EAdi multiplied by respiratory rate. We calculated RSBI as RR divided by VT.

Changes in conventional and EAdi-derived parameters were defined as the difference between the value at each time point of SBT and the value at PS 10. An experienced intensivist carried out diaphragm ultrasound at PS 10 and 30 min after the initiation of SBT. We assessed diaphragm excursion using a low-frequency phased array probe and measured diaphragm thickness during inspiration and expiration using a high-frequency linear probe (Vivid, GE Healthcare, Horten, Norway). Diaphragm excursion and diaphragm thickening fraction were measured three times at PS 10 and 30 min after initial of the SBT. We calculated an average value of the measurements. Diaphragm thickening fraction (DTF) was calculated as (inspiratory thickness-expiratory thickness)/ expiratory thickness. Neuro-excursion efficiency (NEE) was calculated as diaphragm excursion divided by EAdi. Arterial blood gases were collected before SBT and at the end of the SBT before reinstitution of ventilator support.

### Statistical analysis

The minimum sample size was calculated for the diagnostic study using Raosoft sample size calculator. Considering the alpha value of 0.05, power of 80% and the expected sensitivity and specificity to be 0.85 ± 0.1, a total of 35 patients was required [24].

We represented continuous variables as mean ± standard deviation and categorical variables as absolute values and percentages. Shapiro-Wilk normality test was performed to check the distributional characteristics of data. Wilcoxon rank-sum test or Chi-test was used to determine group differences. Given that data distribution was non-normal, Friedman test was used to compare the variance of each time-point within the group. For each tested predictive variable, we calculated areas under the receiver operating characteristics (AUROCs), sensitivity, and specificity to determine the value of predicting weaning outcome. We determined the best cut-off value by computing the Youden index. Correlation analysis was performed using Spearman correlation. For all tests, we considered a two-tailed P value less than 0.05 as significant. IBM SPSS software package version 21.0 (IBM, Armonk, New York) and MedCalc 19.5.6 (MedCalc Software, Mariakerke, Belgium) were used for statistical analysis.

## Results

### Baseline characteristics

A total of 35 were enrolled, with a mean age of 56 ± 16 years. Eighteen (52%) patients were male. The clinical characteristics of enrolled patients were shown in Table 1 and S2. Of the thirty-five patients, thirteen patients experienced failure weaning, with ten patients failing the SBT and three patients requiring reintubated. Pneumonia was the most common primary diagnosis for ICU admission. The SBT failure group had a significantly greater number of ventilation days, more ventilation days before the first SBT, and longer ICU stay compared to the SBT success group. No statistically significant differences were observed between the two groups with respect to other clinical characteristics. Multivariate analysis did not reveal any significant potential confounding factors affecting weaning outcome except for EAdi (Table S3).

**Table 1:** Baseline characteristics of all patients enrolled

	All patients(*n* = 35)	SBT Success(*n* = 25)	SBT Failure(*n* = 10)	*P* value
Age, years	56 ± 16	58 ± 16	51 ± 15	0.25
Male, n (%)	18 (51)	13 (52)	5 (50)	0.92
Body mass index, kg/m^− 2^	25 ± 4	26 ± 4	24 ± 4	0.33
Charlson Comorbidity Index	2 ± 2	2 ± 2	2 ± 2	0.79
Comorbidities (Charlson), n (%)				
Cancer	7 (20)	5 (25)	2 (20)	>0.99
Congestive heart failure	10 (29)	6 (24)	4 (40)	0.83
Myocardial infarction	4 (11)	3 (12)	1 (10)	0.34
Chronic pulmonary disease	9 (26)	7 (28)	2 (20)	0.63
Diabetes	9 (26)	7 (28)	2 (20)	0.63
Liver disease	3 (9)	3 (12)	0 (0)	0.25
Renal disease	3 (9)	2 (8)	1 (10)	0.85
Peripheral vascular disease	5 (14)	2 (8)	3 (30)	0.09
Cerebrovascular disease	3 (9)	2 (8)	1 (10)	0.85
Primary diagnoses for ICU admission, n (%)				
Pneumonia	18 (51)	10 (40)	8 (80)	0.56
Acute heart failure	2 (6)	2 (9)	0 (0)	0.36
Septic shock	5 (14)	4 (16)	1 (10)	0.65
Neurological disorders	7 (20)	6 (24)	1 (10)	0.35
Others	3 (9)	3 (14)	0 (0)	0.25
Modified Barthel Index	4 ± 1	4 ± 1	4 ± 1	0.61
APACHE II score	22 ± 9	22 ± 9	28 ± 9	0.99
SOFA score	9 ± 4	8 ± 4	9 ± 4	0.53
Sepsis, n (%)	27 (77)	18 (72)	9 (90)	0.25
Shock, n (%)	17 (49)	10 (40)	7 (70)	0.11
Corticosteroids treatment, n (%)	21 (60)	14 (56)	7 (70)	0.45
Neuromuscular blockade treatment, n (%)	7 (20)	5 (20)	2 (20)	>0.99
Sedatives treatment, n (%)	35 (100)	25 (100)	10 (100)	-
Controlled ventilation days	3 ± 4	3 ± 3	5 ± 6	0.10
Outcome				
Ventilation days before first SBT	11 ± 7	9 ± 6	15 ± 8	0.02
Ventilation days	20 ± 18	15 ± 12	33 ± 25	0.01
ICU length-of-stays	28 ± 25	21 ± 19	43 ± 34	0.02
Hospital length-of-stays	44 ± 32	42 ± 33	49 ± 34	0.56
28-day mortality, n (%)	7 (20)	4 (16)	3 (30)	0.35

Categorical variables are expressed as absolute value (%) and continuous variables as mean ± SD

ICU intensive care unit; APACHE Acute Physiology and Chronic Health Evaluation; SOFA Sequential Organ Failure Assessment; *P P* value for comparison between the 2 studied groups

### Respiratory parameters at PS 10 cmH_2_O

Table 2 showed the description of SBT success group and failure group at PS 10 cmH_2_O. The SBT success group had a lower average EAdi of 7 ± 3µV, whereas the SBT failure group had an average EAdi of 11 ± 4µV. All EAdi-based parameters indicated a statistically significant difference between the two groups. The success group had lower EAdi, EAdi_AUC_, neuro-discharge per min, but higher neuro-ventilatory efficiency, and neuro-excursion efficiency compared with the failure group (*p* < 0.01). The success group also showed a higher diaphragm excursion (*p* < 0.05). However, there were no significant differences between the two groups at baseline regarding other respiratory parameters.

**Table 2:** Comparison of respiratory parameters between groups and predictability of weaning failure before the SBT

	SBT Success(*n* = 25)	SBT Failure(*n* = 10)	*P* value	
VT, ml	562 ± 167	528 ± 114	0.54	
RR, min^− 1^	17 ± 4	18 ± 4	0.64	
PIP, cmH_2_O	14 ± 1	14 ± 1	0.2	
Pmean, cmH_2_O	7 ± 1	7 ± 1	0.26	
MV, L/min	9 ± 2	9 ± 2	0.78	
Ti/To	0.28 ± 0.06	0.29 ± 0.09	0.92	
P 0.1, cmH_2_O	1.1 ± 0.5	1.4 ± 0.8	0.26	
RSBI, (min·L) ^−1^	35 ± 19	35 ± 13	0.99	
EAdi, µV	7 ± 3	11 ± 4	0.01	
EAdi_AUC_, µV*s	3 ± 1	5 ± 2	0.03	
Neuro-ventilatory efficiency, ml/µV	90 ± 66	51 ± 17	0.01	
Neuro-discharge per min, µV/min	127 ± 65	202 ± 965	0.01	
Neuro-excursion efficiency, cm/µV	0.30 ± 0.28	0.12 ± 0.06	<0.01	
DTF /EAdi, 100%/µV	4.0 ± 2.1	2.0 ± 1.0	<0.01	
Diaphragmatic excursion, cm	1.6 ± 0.6	1.2 ± 0.5	0.04	
Diaphragm thickening fraction, %	25 ± 6	19 ± 9	0.07	
End-expiratory thickness of diaphragm, cm	0.43 ± 0.15	0.36 ± 0.13	0.19	

VT tidal volume; RR respiratory rate; PIP peak inspiratory pressure; Pmean mean airway pressure; MV minute ventilation volume; Ti/To inspiratory time/ total respiratory cycle time; P 0.1 airway occlusion pressure at 100 ms; RSBI rapid shallow breathing index, calculated as RR divided by VT; EAdi electrical activity of the diaphragm, calculated as maximum EAdi during inspiration minus EAdi minimum; EAdi_AUC_ area under the curve of EAdi signal over time from its onset to its peak value; Neuro-ventilatory efficiency, calculated as VT divided by EAdi; Neuro-discharge per minute, calculated as RR multiplied by EAdi; Neuro-excursion efficiency, calculated as diaphragm excursion divided by EAdi; DTF Diaphragm thickening fraction, calculated as (inspiratory thickness-expiratory thickness)/ expiratory thickness*100%; *P P* value for comparison between the 2 studied groups; AUROC area under the receiver operating characteristic; CI confidence interval

### Predictability of respiratory parameters at PS 10 cmH_2_O

**Fig. 1:**
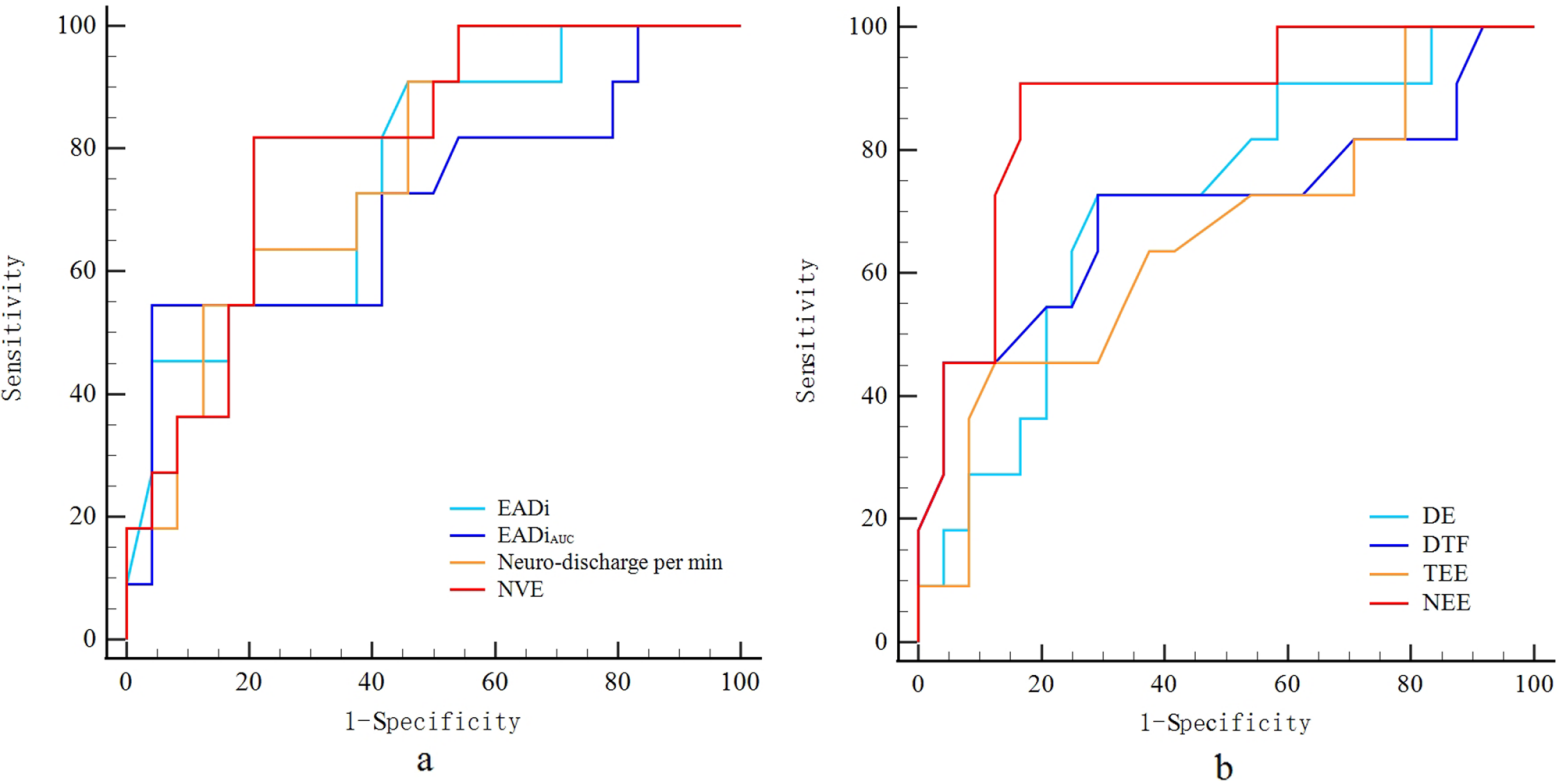
Receiver operating characteristic curves to predict SBT outcome at PS 10 cmH_2_O. EAdi electrical activity of the diaphragm, calculated as maximum EAdi during inspiration minus EAdi minimum; EAdi_AUC_ area under the curve of EAdi signal over time from its onset to its peak value; Neuro-discharge per min, calculated as RR multiplied by EAdi; NVE Neuro-ventilatory efficiency, calculated as VT divided by EAdi; DE diaphragmatic excursion; DTF diaphragm thickening fraction; TEE, end-expiratory thickness of diaphragm; NEE neuro-excursion efficiency, calculated as diaphragm excursion divided by EAdi

At PS 10 cmH_2_O, the EAdi and EAdi_AUC_ had AUROCs of 0.763 and 0.710, respectively, for predicting SBT failure. EAdi-derived parameters demonstrated excellent predictive ability. Neuro-ventilatory efficiency and neuro-discharge per min had AUROCs of 0.807 and 0.777, respectively, while neuro-excursion efficiency had the best predictive ability with an AUROC of 0.875. However, the ultrasonic index including diaphragm excursion, thickening fraction, and end-expiratory thickness of diaphragm had only moderate or poor predictive ability (Fig. 1).

### Temporal trend of parameters during the SBT

**Fig. 2:**
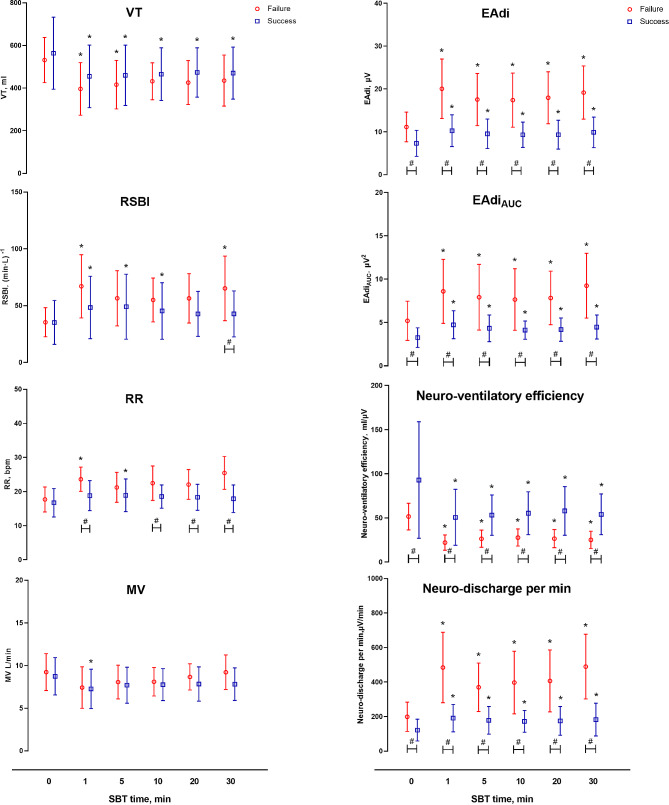
Conventional parameters and EAdi-based parameters before and during the SBT (1, 5, 10, 20 and 30 min) in patients in patients successfully passed the SBT (square) and failed the SBT (circle). VT tidal volume; RR respiratory rate; RSBI rapid shallow breathing index, calculated as RR divided by VT; MV minute ventilation volume; EAdi electrical activity of the diaphragm, calculated as maximum EAdi during inspiration minus EAdi minimum; EAdi_AUC_ area under the curve of EAdi signal over time from its onset to its peak value; Neuro-ventilatory efficiency, calculated as VT divided by EAdi; Neuro-discharge per min, calculated as RR multiplied by EAdi. * *P*<0.05 versus PS 10 cmH_2_O(SBT 0 min)within each group, # *P*<0.05 in comparison to the same time between two groups; AUROC area under the receiver operating characteristic; CI confidence interval

Figure 2 illustrates the temporal trend of parameters during the SBT. Significant changes in VT, RR, and RSBI were observed in both groups at the partial time point compared to PS 10 cmH_2_O. After initiation of the SBT trial, RR and RSBI rapidly increased in both groups. During the rest of the SBT, RR and RSBI gradually increased in the failure group and remained stable in the success group. Significant differences were observed between the two groups in the RSBI at the 30-minute mark of the SBT, and in the RR at 1, 10, 20, and 30-minute marks. Traditional indicators showed no continuous inter-group differences during the SBT. After removing the PS, EAdi increased from approximately 11 to 20 µV in the failure group and from 7 to 10 µV in the success group within a minute. From the 1-minute mark to the 30-minute mark of the SBT, EAdi increased in the failure group and remained stable in the success group. Similar trends were observed in neuro-discharge per min, with the changes in the failure group being more significant. All EAdi-based parameters showed significant differences between the two groups (*p* < 0.05).

**Fig. 3:**
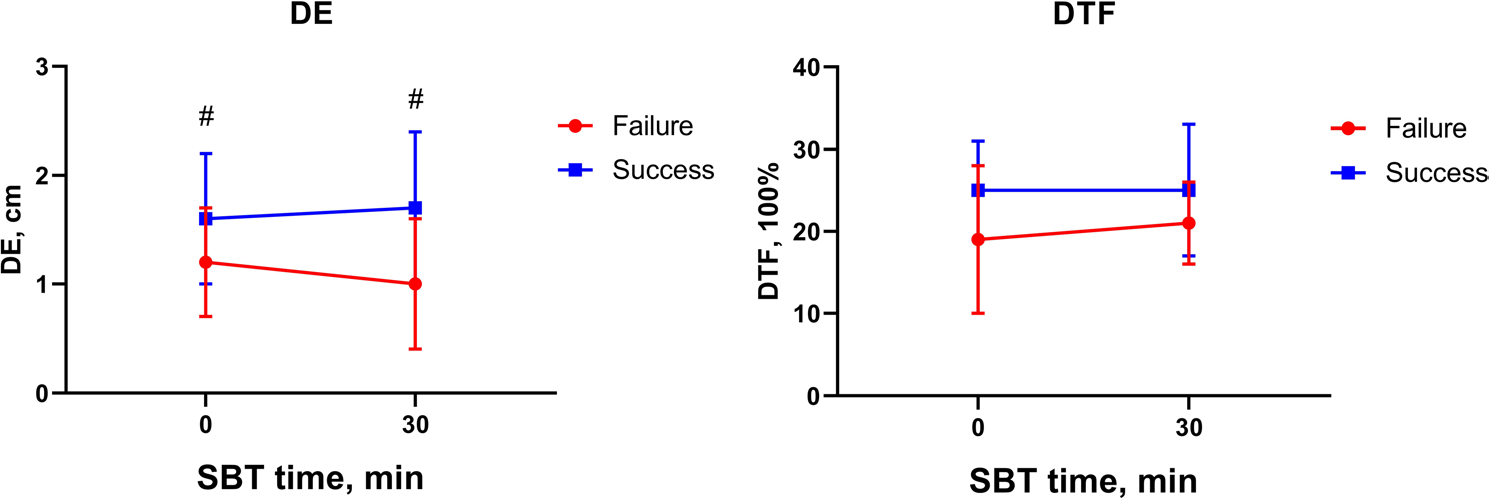
Diaphragm ultrasonography parameters before and at the 30 min of the SBT in patients successfully passed the SBT (square) and failed the SBT (circle). DE Diaphragmatic excursion; NEE Neuro-excursion efficiency, calculated as diaphragm excursion divided by EAdi; DTF Diaphragm thickening fraction, calculated as (inspiratory thickness-expiratory thickness)/ expiratory thickness*100%. # *P*<0.05 in comparison to the same time between two groups

Figure 3 illustrates diaphragm ultrasonography parameters before and 30 min after the initial of the SBT. No differences were found in DE and DTF before and at the 30 min of the SBT, independently of the weaning outcome. SBT success groups had significantly more DE than SBT failure group before and at the 30 min of the SBT.

### Predictability of absolute value and change of respiratory parameters for weaning failure during the SBT

**Fig. 4:**
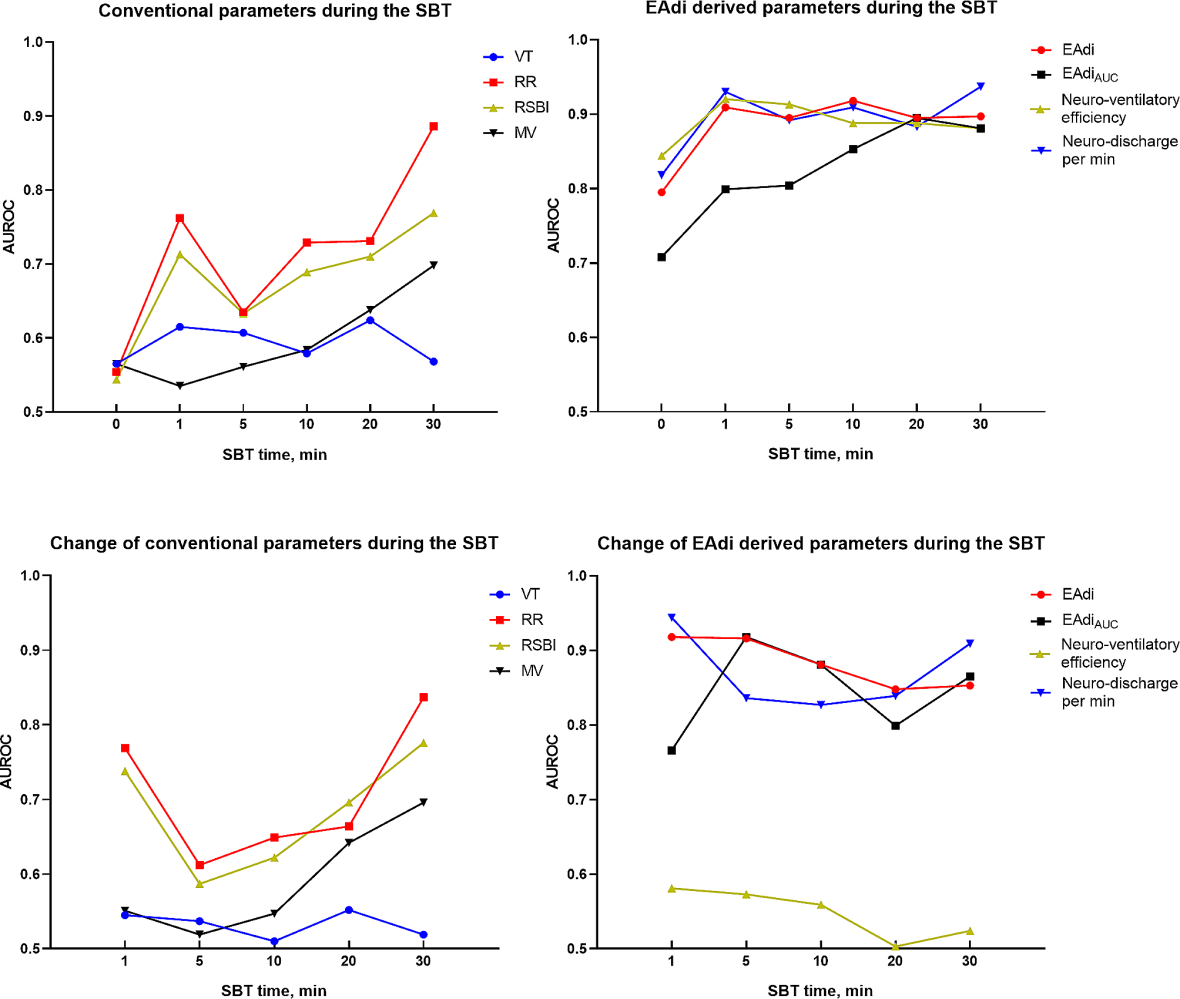
AUROC (area under receiver operating characteristic) for the accuracy of absolute value and change of conventional and EAdi-based parameters from the baseline(SBT 0 min)to predict weaning failure during the SBT. VT tidal volume; RR respiratory rate; RSBI rapid shallow breathing index, calculated as RR divided by VT; MV minute ventilation volume; EAdi electrical activity of the diaphragm, calculated as maximum EAdi during inspiration minus EAdi minimum; EAdi_AUC_ area under the curve of EAdi signal over time from its onset to its peak value; Neuro-ventilatory efficiency, calculated as VT divided by EAdi; Neuro-discharge per min, calculated as RR multiplied by EAdi

Following the removal of pressure support, most parameters exhibited higher predictability compared to the PS 10 cmH_2_O, as illustrated in Fig. 4. Overall, EAdi-based parameters showed better predictability than conventional parameters. During the 30-minute SBT, the AUROCs of RR and RSBI were 0.886 and 0.769, respectively. Throughout the entire SBT, EAdi, neuro-ventilatory efficiency, and neuro-discharge per min presented AUROCs of 0.897–0.918, 0.881–0.920, and 0.883–0.930, respectively. Changes in EAdi and neuro-discharge per min at 1 min were found to provide higher AUROCs of 0.918 and 0.944, respectively. However, changes in neuro-ventilatory efficiency only provide a AUROC of 0.503–0.581. Details of AUROC values, cut-off points, specificity, and sensitivity for each parameter at each time point were shown in the Table S4–S7.

## Discussion

In this prospective study, we were able to describe the physiological behavior and explore the role evaluate the possibility of weaning success. Several major findings were observed. First, the EAdi-based parameters exhibited significant differences between the SBT success group and failure group. Second, in patients with SBT failure, the changes of EAdi parameters in SBT were more significant than diaphragmatic ultrasound and conventional respiratory parameters, especially in SBT failure group. Third, early changes of EAdi-based parameters during the SBT showed excellent predictability for weaning failure. Neuro-excursion efficiency was the best predictor at the PS 10 and the change of neuro-discharge per min at 1 min after SBT initiation was the best predictor during the SBT.

To our knowledge, this study is the first report on the feasibility of combined EAdi parameters and diaphragm ultrasound in weaning patients. Neuro-excursion efficiency was employed to evaluate the readiness of weaning before SBT trials were implemented. This parameter was able to reflect the ability of the diaphragm to convert respiratory drive into diaphragmatic movement. In previous studies, other parameters were used to convert respiratory neural drive into ventilation, such as neuro-ventilatory efficiency and neuro-mechanical efficiency, which respectively used VT and the decrease in airway pressure during inspiratory effort, divided by EAdi [12, 18, 25]. However, VT and inspiratory effort are influenced by accessory inspiratory muscles besides the diaphragm, which could not represent the efficiency of the direct conversion from the phrenic nerve to phrenic muscle [26]. In our patients, the AUROC of neuro-ventilatory efficiency for weaning outcome was 0.844, but neuro-excursion efficiency exhibited the best predictive value (AUROC 0.874) before the weaning initiation. In addition, neuro-mechanical efficiency was not measured because repeated measurements of neuro-mechanical efficiency showed high variability, limiting the ability of a single action to estimate neuromuscular efficiency [25].

While our study and Dres’s study revealed a baseline difference in EAdi, other studies have failed to demonstrate such variations [16, 17, 27, 28]. We hypothesized that the increased neural drive in the failure group was insignificant during ventilator assistance but became significant upon the withdrawal of ventilator support. Barwing and colleagues reported a significant increase in EAdi during weaning failure using a standard SBT protocol [16]. These changes in EAdi, EAdi_AUC_, and EAdi/VT were also found in Dres’s study [17]. However, the potential value of this early change in predicting weaning outcomes has never been investigated. We chose to collect data one minute after starting the SBT to assess the immediate compensation of patients’ neural drive following the removal of ventilatory support. As anticipated, our results suggested that monitoring the early changes in EAdi and neuro-discharge per min during SBT might help predict weaning outcomes.

At PS 10, EAdi showed a significant difference between groups, whereas RR and RSBI did not. This suggested that EAdi may detect potential dyspnea that cannot be clinically recognized through fast shallow breathing. During the SBT, EAdi also exhibited more differences than conventional parameters in the failure group. Barwing and colleagues continuously monitored EAdi during the SBT and found that patients who failed the SBT experienced an earlier increase in EAdi than in RR and VT [16]. In our study, we found that the AUROC of RR and VT gradually increased and reached a maximum at 30 min of the SBT. However, EAdi-based parameters achieved better predictivity in the early stages of the SBT. These findings might aid in understanding the physiological processes of respiratory drive. When the inspiratory muscle strength is unable to match the respiratory system’s burden, the respiratory drive increases to meet the ventilation as much as possible. When the respiratory drive increases to a certain extent, a decrease in VT and compensatory RR is shown.

Diaphragmatic ultrasound is increasingly used to assess the readiness for weaning from mechanical ventilation [11, 29]. Nevertheless, the predictability for weaning failure and the utility of diaphragmatic ultrasound in the SBT are still controversial [10, 30]. In SBT failure group, the diaphragmatic ultrasound indexes did not change significantly before and after 30-min SBT, but the EAdi-based parameters significantly change, suggesting that EAdi was more suitable for SBT monitoring. It can be also speculated that the increase in neural drive appeared earlier than diaphragm fatigue. Our study also confirmed the better predictive performance of EAdi-based parameters for SBT failure. In a recent study, Tayar and Abdelshafey found that diaphragmatic ultrasound measurements were more useful than EAdi parameters in predicting liberation outcome [28], which contradicted our results. Our study produced AUROCs of 0.714 and 0.703 for diaphragm excursion and thickening fraction, respectively, which were comparable to the results of a previous meta-analysis [10]. However, diaphragm dysfunction alone did not necessarily indicate weaning failure, as other factors like pulmonary disorders and heart failure might also influence weaning outcome [31]. Furthermore, the conflicting findings might be related to the study population, as patients with severe neuromuscular diseases such as motor neuron disease might present with normal or low EAdi and obvious diaphragm dysfunction [32]. Importantly, reliability of ultrasound-depending parameters might be influenced by intra- and inter-investigator variability but EAdi might be more stable and continuous real-time monitoring of EAdi would be a major advantage. We therefore recommend EAdi-based parameters. While diaphragmatic ultrasound was not a reliable predictor of weaning outcome, it could still aid in the assessment of diaphragm function and identification of the underlying cause of weaning failure.

The optimal duration of SBT is still controversy. Studies have shown that 120-min SBT presented no difference with 30-min SBT in weaning failure rate [33, 34]. Conversely, a large scale RCT recently showed that 30-min SBT has been considered to led to significantly higher rates of successful extubation [35]. Vallverdú et al. found that among patients failing a 2-hour T-piece SBT, 36% failed occurred between 30 min and 2 h [35]. Evidence has been presented that patients with SBT failures after 30 min were older, had more cardiopulmonary disease than patients with SBT failures before the initial 30 min. Taking our patients’ characteristics into account, 2-h SBT was selected for weaning evaluation in this study. CPAP as selected for weaning evaluation because removal of PS could be used to evaluated patients’ compensatory ability to withdrawal of pressure support and tidal volume could be monitored during the SBT. The present studies suggested that CPAP was not inferior to PS and t-piece [36, 37].

In this study, the failure group had a significantly greater number of ventilation days, ventilation days before the first SBT, and length of ICU stay compared to the success group. Spearman correlation analysis showed there is no correlation between EAdi and ventilation days before the first SBT (*P*>0.05) (Table S9). Neuro-excursion efficiency and diaphragm excursion at PS 10 had a significant correlation with ventilation days before the first SBT (*P*<0.05). We therefore speculated that prolonged mechanical ventilation affects the conversion efficiency mainly by impairing the diaphragm function, as shown in previous studies [29, 38].

Our study had several limitations. First, it was an open-label study, which leaves it vulnerable to performance and detection biases. Second, the study population was small and confined to a single center, making it difficult to generalize the predictive value of certain parameters and introducing sample bias. Further external validation is warranted. Third, we did not conduct subgroup analysis for different diseases, despite the possibility of differing pathophysiological mechanisms of weaning failure. Although we included patients with various diseases, further data is needed to assess the predictive value of EAdi-based parameters in different populations. Besides, the percentage of weaning failure might be low because PS were not used to compensate for the resistance of the tracheal intubation. Lastly, neuro-excursion efficiency was measured with PS and part of the diaphragm excursion is due to positive pressure ventilation. Umbrello suggested patients should be briefly disconnected for the evaluation of diaphragm excursion [39]. However, the contraction of the diaphragm after disconnection is not stable and might lead to measurement bias.

## Conclusion

Patients with failed weaning showed reduced efficiency in converting neural drive into muscle force. Early increases in EAdi occur in SBT, which are more prominent in SBT failure group. Our study supported the potential use of monitoring EAdi-based parameters before and during the SBT to predict weaning outcomes. Larger studies are needed to validate our findings and develop standardized protocols for monitoring EAdi in clinical settings.

### Electronic supplementary material

Below is the link to the electronic supplementary material.

Supplementary Material 1

## Data Availability

The datasets and materials analyzed during the current study are available from the corresponding author on reasonable request.

## Declarations

### Generative AI in scientific writing

No AI tools were used as part of the research process.
