# Supplementary material for: The diaphragmatic electrical activity during spontaneous breathing trial in patients with mechanical ventilation: physiological description and potential clinical utility
Source: BMC Pulm Med. 2024 May 30;24:263. doi: 10.1186/s12890-024-03077-8 (PMC11140881; doi:10.1186/s12890-024-03077-8)
Supplement: Supplementary file 1 — Supplementary Material 1 [file 12890_2024_3077_MOESM1_ESM.docx]

**Supplementary Materials**

**Content**

[Table S1 Details for positioning the EAdi catheter and checking positions. 2](#_Toc143718124)

[Table S2 Detailed patient data at baseline. 3](#_Toc143718125)

[Table S3 Binary logistic regression analysis for weaning outcome 5](#_Toc143718126)

[Table S4 Weaning predictability of electrical activity of the diaphragm (EAdi)-based parameters during the spontaneous breathing trial. 6](#_Toc143718127)

[Table S5 Weaning predictability of change of EAdi-based parameters during the spontaneous breathing trial. 8](#_Toc143718128)

[Table S6 Weaning predictability of conventional parameters during the spontaneous breathing trial. 10](#_Toc143718129)

[Table S7 Weaning predictability of change of conventional parameters during the spontaneous breathing trial. 12](#_Toc143718130)

[Table S8 Arterial blood gas before and after the SBT. 13](#_Toc143718131)

[Table S9 Spearman correlation analysis for Ventilation days before first SBT 14](#_Toc143718132)

# Table S1 Details for positioning the EAdi catheter and checking positions.

| 1. Patients were in supine position with the upper part of the body elevated in a 30° angle and gastric content was drained via the nasogastric tube. Afterwards the standard tubing was replaced by the EAdi-catheter (16 f, 125 cm long; Maquet Critical Care, Solna, Sweden), inserted nasally to a maximum distance of 80 cm. |
| --- |
| 1. We calculated the expected insertion distance of the EAdi-catheter (NEX)   NEX_mod_=NEX*0.9+18(for the 16f. EAdi-catheter) |
| 1. The EAdi-catheter was placed at a depth of more than 5 cm NEX. Successful placement of the gastric insufflation was confirmed by air insufflation and epigastric auscultation and by aspiration of gastric juice using vacuum suction. |
| 1. Catheter positioning was monitored by a special tool implemented in the ventilator. It displays an EAdi curve and four raw leads not filtered for ECG activity. The electrical activity used for generating the EAdi signal is highlighted. The position of the electrodes in relation to the heart and diaphragm can be estimated by evaluating the different leads for presence/absence of p-wave and QRS complex. During the placement procedure the catheter was pulled out in steps of 1 cm. EAdi signal and electrical activity from raw leads were recorded at each step using special software (NAVA tracker, Maquet Critical Care, Solna, Sweden) for offline analysis until ECG-signals disappeared. The “optimal” catheter position (OPT) was identified by checking the recording for the following three criteria: (1) stable EAdi signal, (2) electrical activity highlighted in central leads of the catheter positioning tool, and (3) absence of p-wave in distal lead. Within the catheter positions that fulfilled the above mentioned criteria we chose the one with the maximum EAdi value as OPT. |
| 1. The EAdi-catheter was properly fixed with the pressure tape. |

# Table S2 Detailed patient data at baseline.

| No. | F/M | Age | BMI  (kg/m^-2^) | Weaning outcome | Primary diagnosis | Cause of weaning failure | ICU d | APACHEⅡ | SOFA | EAdi  （μV） | RSBI  (min·L ^-1^) | DE  (cm) |
| --- | --- | --- | --- | --- | --- | --- | --- | --- | --- | --- | --- | --- |
| 1 | M | 54 | 22 | F 0-30 | Pancreatitis | L | 135 | 17 | 10 | 7.9 | 49 | 0.34 |
| 2 | F | 76 | 26 | S | Intracranial infection | - | 22 | 45 | 13 | 15.9 | 44 | 0.93 |
| 3 | M | 63 | 23 | R | Pompe's disease | D | 35 | 25 | 7 | 15.9 | 56 | 0.69 |
| 4 | F | 37 | 20 | F 0-30 | Pneumonia | L | 24 | 28 | 10 | 16.6 | 34 | 1.08 |
| 5 | F | 65 | 21 | S | Pneumonia | - | 21 | 24 | 4 | 8.9 | 19 | 2.78 |
| 6 | F | 46 | 24 | S | Septic shock | - | 26 | 20 | 11 | 2.6 | 57 | 1.02 |
| 7 | F | 76 | 26 | F 0-30 | Pneumonia | C | 48 | 37 | 15 | 7.5 | 28 | 1.18 |
| 8 | F | 58 | 23 | S | Septic shock | - | 10 | 15 | 8 | 9.9 | 18 | 0.91 |
| 9 | M | 48 | 28 | S | Myocardial infarction | - | 72 | 25 | 9 | 7.4 | 22 | 1.53 |
| 10 | F | 90 | 30 | S | Pneumonia | - | 42 | 19 | 8 | 4.9 | 44 | 1.15 |
| 11 | F | 66 | 22 | S | Cardiac arrest | - | 18 | 41 | 13 | 4.5 | 28 | 1.7 |
| 12 | F | 55 | 18 | F 0-30 | Pneumonia | L | 37 | 17 | 7 | 8.8 | 22 | 0.54 |
| 13 | M | 69 | 22 | R | Septic shock | A | 40 | 21 | 10 | 11.3 | 21 | 0.4 |
| 14 | M | 20 | 24 | F 0-30 | Metabolic myopathy | D | 40 | 8 | 1 | 14.8 | 25 | 1.13 |
| 15 | F | 26 | 35 | S | Meningitis | - | 4 | 15 | 4 | 4.8 | 61 | 1.76 |
| 16 | M | 45 | 22 | S | Diabetic ketoacidosis | - | 11 | 26 | 7 | 6.5 | 88 | 0.56 |
| 17 | M | 47 | 23 | S | Toxic encephalopathy | - | 9 | 22 | 16 | 2 | 20 | 3.1 |
| 18 | M | 59 | 28 | S | Alveolar hemorrhage | - | 10 | 26 | 12 | 6.3 | 22 | 1.8 |
| 19 | F | 29 | 20 | S | Neurosyphilis | - | 7 | 8 | 4 | 5.8 | 15 | 1.55 |
| 20 | F | 59 | 25 | F 0-30 | Pneumonia | L | 12 | 22 | 7 | 8.5 | 27 | 2.09 |
| 21 | M | 43 | 31 | S | Pneumonia | - | 14 | 9 | 9 | 9.6 | 8 | 2.1 |
| 22 | F | 78 | 31 | S | Meningitis | - | 9 | 40 | 10 | 11 | 16 | 1.72 |
| 23 | M | 65 | 24 | S | Pulmonary fibrosis | - | 8 | 15 | 8 | 10.8 | 30 | 2 |
| 24 | M | 71 | 23 | S | Septic shock | - | 11 | 24 | 12 | 5 | 27 | 1.5 |
| 25 | M | 35 | 35 | S | Pneumonia | - | 17 | 21 | 12 | 8.4 | 39 | 2.4 |
| 26 | M | 50 | 22 | F 0-30 | Pneumonia | L | 38 | 31 | 15 | 10.7 | 25 | 1.3 |
| 27 | F | 51 | 28 | S | Pneumonia | - | 10 | 19 | 6 | 6.2 | 71 | 1.3 |
| 28 | F | 74 | 24 | S | Pneumonia | - | 5 | 23 | 4 | 9.4 | 29 | 1.93 |
| 29 | F | 34 | 23 | S | Pneumonia | - | 5 | 16 | 2 | 6.6 | 40 | 1.8 |
| 30 | M | 76 | 25 | R | Pneumonia | D | 41 | 13 | 4 | 9.2 | 50 | 1.7 |
| 31 | M | 60 | 23 | S | Pneumonia | - | 18 | 10 | 8 | 7.5 | 37 | 1.8 |
| 32 | M | 69 | 29 | S | Pneumonia | - | 70 | 22 | 7 | 7.3 | 36 | 1.37 |
| 33 | F | 67 | 27 | F 30-120 | Pneumonia | C | 21 | 25 | 8 | 6 | 57 | 1 |
| 34 | M | 51 | 28 | F 30-120 | Pneumonia | L | 50 | 14 | 10 | 15.9 | 41 | 1.75 |
| 35 | M | 36 | 30 | F 0-30 | Pneumonia | L | 28 | 19 | 9 | 11.7 | 24 | 1.7 |

F/M Female/Male; BMI Body mass index; Weaning outcome F 0-30 SBT failed at 0-30 minutes, F 30-120 SBT failed at 30-120 minutes, R reintubation, S weaning success, Cause of weaning failure A Airway, L Lung dysfunction, C Cardiac dysfunction, D Diaphragm/respiratory muscle function; EAdi electrical activity of the diaphragm；RSBI Rapid shallow breathing index; DE Diaphragmatic excursion.

# Table S3 Binary logistic regression analysis for weaning outcome

|  | P | OR | 95%CI |
| --- | --- | --- | --- |
| Ventilation days before first SBT | 0.07 | 0.89 | 0.81-1.04 |
| EAdi | 0.02 | 0.86 | 0.68-1.08 |
| Diaphragm excursion | 0.18 | 0.28 | 0.05-1.53 |

# Table S4 Weaning predictability of electrical activity of the diaphragm (EAdi)-based parameters during the spontaneous breathing trial.

|  | AUROC (95%CI) | Cut-off value | Sensitivity（%） | Specificity（%） |
| --- | --- | --- | --- | --- |
| EAdi-0min | 0.795 (0.625 - 0.913) | 7.4 | 59.1 | 92.3 |
| EAdi-1min | 0.909 (0.763 - 0.980) | 13.1 | 86.4 | 92.3 |
| EAdi-5min | 0.895 (0.745 - 0.973) | 11.7 | 86.4 | 92.3 |
| EAdi-10min | 0.918 (0.774 - 0.984) | 12.1 | 95.4 | 92.3 |
| EAdi-20min | 0.895 (0.745 - 0.973) | 10.8 | 86.4 | 92.3 |
| EAdi-30min | 0.897 (0.747 - 0.974) | 14 | 90.9 | 84.6 |
| EAdi_AUC_-0min | 0.708 (0.530 - 0.849) | 5 | 100 | 53.9 |
| EAdi_AUC_-1min | 0.799 (0.629 - 0.915) | 8.9 | 100 | 61.5 |
| EAdi_AUC_-5min | 0.804 (0.635 - 0.918) | 4.6 | 62.2 | 84.6 |
| EAdi_AUC_-10min | 0.853 (0.693 - 0.950) | 5.4 | 95.5 | 76.9 |
| EAdi_AUC_-20min | 0.895 (0.745 - 0.973) | 10.8 | 86.4 | 92.3 |
| EAdi_AUC_-30min | 0.881 (0.727 - 0.966) | 6.6 | 95.5 | 76.9 |
| Neuro-discharge per min-0min | 0.818 (0.651 - 0.928) | 150.4 | 86.4 | 69.2 |
| Neuro-discharge per min-1min | 0.930 (0.791 - 0.989) | 291.9 | 95.5 | 84.6 |
| Neuro-discharge per min-5min | 0.892 (0.740 - 0.971) | 266.8 | 90.9 | 84.6 |
| Neuro-discharge per min-10min | 0.909 (0.763 - 0.980) | 233.2 | 90.9 | 84.6 |
| Neuro-discharge per min-20min | 0.883 (0.729 - 0.966) | 312.9 | 95.4 | 69.2 |
| Neuro-discharge per min-30min | 0.937 (0.800 - 0.991) | 216 | 72.7 | 100 |
| Neuro-ventilatory efficiency-0min | 0.844 (0.682 - 0.944) | 58.9 | 86.4 | 84.6 |
| Neuro-ventilatory efficiency-1min | 0.920 (0.776 - 0.984) | 34.4 | 81.8 | 92.3 |
| Neuro-ventilatory efficiency-5min | 0.913 (0.767 - 0.981) | 42.4 | 77.3 | 100 |
| Neuro-ventilatory efficiency-10min | 0.888 (0.736 - 0.969) | 41.0 | 77.3 | 92.3 |
| Neuro-ventilatory efficiency-20min | 0.888 (0.736 - 0.969) | 43.0 | 77.3 | 92.3 |
| Neuro-ventilatory efficiency-30min | 0.881 (0.727 - 0.966) | 30.5 | 86.4 | 84.6 |
| Neuro-excursion efficiency-0min | 0.874 (0.718 - 0.962) | 0.185 | 81.8 | 92.3 |
| Neuro-excursion efficiency-30min | 0.874 (0.718 - 0.962) | 0.105 | 86.4 | 84.6 |

EAdi electrical activity of the diaphragm, calculated as maximum EAdi during inspiration minus EAdi minimum; EAdi_AUC_ area under the curve of EAdi signal over time from its onset to its peak value; Neuro-ventilatory efficiency, calculated as VT divided by EAdi; Neuro-discharge per min calculated as RR multiplied by EAdi; AUROC area under the receiver operating characteristic; CI confidence interval. The cutoff values for indicators were determined by ROC analyses (Youden Index).

# Table S5 Weaning predictability of change of EAdi-based parameters during the spontaneous breathing trial.

|  | AUROC (95%CI) | Cut-off value | Sensitivity（%） | Specificity（%） |
| --- | --- | --- | --- | --- |
| EAdi-1min | 0.918 (0.774 - 0.984) | 3.9 | 81.8 | 92.3 |
| EAdi-5min | 0.916 (0.772 - 0.983) | 4.9 | 100 | 76.9 |
| EAdi-10min | 0.881 (0.727 - 0.966) | 4.2 | 100 | 76.9 |
| EAdi-20min | 0.848 (0.686 - 0.946) | 5.3 | 90.9 | 69.2 |
| EAdi-30min | 0.853 (0.693 - 0.950) | 6 | 90.9 | 76.9 |
| EAdi_AUC_-1min | 0.766 (0.592 - 0.892) | 2.7 | 95.5 | 61.5 |
| EAdi_AUC_-5min | 0.918 (0.774 - 0.984) | 4.9 | 100 | 76.9 |
| EAdi_AUC_-10min | 0.881 (0.727 - 0.966) | 4.2 | 100 | 76.9 |
| EAdi_AUC_-20min | 0.799 (0.629 - 0.915) | 1.5 | 77.3 | 84.6 |
| EAdi_AUC_-30min | 0.865 (0.707 - 0.957) | 1.8 | 81.8 | 84.6 |
| Neuro-discharge per min-1min | 0.944 (0.810 - 0.993) | 123.9 | 95.5 | 92.3 |
| Neuro-discharge per min-5min | 0.836 (0.672 - 0.939) | 117.8 | 95.5 | 69.2 |
| Neuro-discharge per min-10min | 0.827 (0.662 - 0.933) | 87 | 95.5 | 76.9 |
| Neuro-discharge per min-20min | 0.839 (0.676 - 0.941) | 113.2 | 86.4 | 76.9 |
| Neuro-discharge per min-30min | 0.909 (0.763 - 0.980) | 101.4 | 77.3 | 92.3 |
| Neuro-ventilatory efficiency-1min | 0.581 (0.409 - 0.751) | -47.9 | 36.4 | 92.3 |
| Neuro-ventilatory efficiency-5min | 0.573 (0.395 - 0.739) | -39.7 | 40.9 | 92.3 |
| Neuro-ventilatory efficiency-10min | 0.559 (0.382 - 0.726) | -36.0 | 31.8 | 92.3 |
| Neuro-ventilatory efficiency-20min | 0.503 (0.330 - 0.676) | -33.5 | 72.7 | 7.7 |
| Neuro-ventilatory efficiency-30min | 0.524 (0.349 - 0.695) | -27.2 | 54.6 | 61.5 |

EAdi electrical activity of the diaphragm, calculated as maximum EAdi during inspiration minus EAdi minimum; EAdi_AUC_ area under the curve of EAdi signal over time from its onset to its peak value; Neuro-ventilatory efficiency Neuro-ventilatory efficiency, calculated as VT divided by EAdi; Neuro-discharge per min, calculated as RR multiplied by EAdi; AUROC area under the receiver operating characteristic; CI confidence interval. The cutoff values for indicators were determined by ROC analyses (Youden Index).

# Table S6 Weaning predictability of conventional parameters during the spontaneous breathing trial.

|  | AUROC (95%CI) | Cut-off value | Sensitivity（%） | Specificity（%） |
| --- | --- | --- | --- | --- |
| RR-0min | 0.554 (0.377 - 0.722) | 22 | 95.5 | 23.1 |
| RR-1min | 0.762 (0.589 - 0.889) | 18 | 40.9 | 100 |
| RR-5min | 0.635 (0.455 - 0.790) | 15 | 36.4 | 100 |
| RR-10min | 0.729 (0.553 - 0.865) | 22 | 95.5 | 53.9 |
| RR-20min | 0.731 (0.554 - 0.866) | 18 | 54.6 | 76.9 |
| RR-30min | 0.886 (0.733 - 0.968) | 20 | 77.3 | 84.6 |
| VT-0min | 0.565 (0.387 - 0.731) | 680 | 27.3 | 100 |
| VT-1min | 0.615 (0.436 - 0.774) | 450 | 50.0 | 76.9 |
| VT-5min | 0.607 (0.428 - 0.767) | 454 | 54.5 | 76.9 |
| VT-10min | 0.579 (0.400 - 0.743) | 530 | 31.8 | 92.3 |
| VT-20min | 0.624 (0.445 - 0.782) | 340 | 86.4 | 38.5 |
| VT-30min | 0.568 (0.390 - 0.734) | 580 | 27.3 | 92.3 |
| RSBI-0min | 0.544 (0.367 - 0.712) | 20 | 27.3 | 100 |
| RSBI-1min | 0.713 (0.536 - 0.853) | 47 | 68.2 | 76.9 |
| RSBI-5min | 0.633 (0.454 - 0.789) | 50 | 72.7 | 61.5 |
| RSBI-10min | 0.689 (0.510 - 0.834) | 44 | 72.7 | 69.2 |
| RSBI-20min | 0.710 (0.532 - 0.850) | 44 | 72.7 | 69.2 |
| RSBI-30min | 0.769 (0.596 - 0.894) | 41 | 68.2 | 84.6 |
| MV-0min | 0.565 (0.387 - 0.731) | 8.3 | 54.6 | 76.9 |
| MV-1min | 0.535 (0.359 - 0.705) | 8.2 | 72.7 | 46.2 |
| MV-5min | 0.561 (0.384 - 0.728) | 8.3 | 68.2 | 53.8 |
| MV-10min | 0.584 (0.406 - 0.748) | 6.2 | 36.4 | 92.3 |
| MV-20min | 0.638 (0.459 - 0.793) | 7.8 | 63.6 | 69.2 |
| MV-30min | 0.698 (0.519 - 0.841) | 9.5 | 86.4 | 61.5 |

VT tidal volume; RR respiratory rate; RSBI rapid shallow breathing index, calculated as RR divided by VT; MV minute ventilation volume; AUROC area under the receiver operating characteristic; CI confidence interval. The cutoff values for indicators were determined by ROC analyses (Youden Index).

# Table S7 Weaning predictability of change of conventional parameters during the spontaneous breathing trial.

|  | AUROC (95%CI) | Cut-off value | Sensitivity（%） | Specificity（%） |
| --- | --- | --- | --- | --- |
| RR-1min | 0.769 (0.596 - 0.894) | 5 | 86.4 | 61.5 |
| RR-5min | 0.612 (0.433 - 0.771) | 5 | 86.4 | 38.5 |
| RR-10min | 0.649 (0.469 - 0.802) | 5 | 86.4 | 46.1 |
| RR-20min | 0.664 (0.485 - 0.814) | 3 | 77.3 | 53.9 |
| RR-30min | 0.837 (0.674 - 0.940) | 5 | 90.9 | 69.2 |
| VT-1min | 0.545 (0.369 - 0.714) | -180 | 77.3 | 38.5 |
| VT-5min | 0.537 (0.361 - 0.706) | -40 | 81.8 | 46.2 |
| VT-10min | 0.510 (0.336 - 0.683) | -107 | 68.2 | 53.9 |
| VT-20min | 0.552 (0.375 - 0.720) | -127 | 72.7 | 46.2 |
| VT-30min | 0.519 (0.344 - 0.691) | -140 | 81.8 | 38.5 |
| RSBI-1min | 0.738 (0.562 - 0.871) | 30 | 95.5 | 53.9 |
| RSBI-5min | 0.587 (0.409 - 0.751) | 30 | 90.9 | 38.5 |
| RSBI-10min | 0.622 (0.443 - 0.780) | 25 | 95.5 | 46.2 |
| RSBI-20min | 0.696 (0.518 - 0.839) | 20 | 95.5 | 46.2 |
| RSBI-30min | 0.776 (0.604 - 0.899) | 17 | 86.4 | 76.9 |
| MV-1min | 0.551 (0.374 - 0.719) | -1.7 | 45.5 | 76.9 |
| MV-5min | 0.519 (0.344 - 0.691) | -0.9 | 59.1 | 61.2 |
| MV-10min | 0.547 (0.371 - 0.716) | -1.1 | 63.6 | 61.5 |
| MV-20min | 0.642 (0.462 - 0.796) | -0.4 | 72.7 | 69.2 |
| MV-30min | 0.696 (0.518 - 0.839) | -0.5 | 90.9 | 53.9 |

VT tidal volume; RR respiratory rate; RSBI rapid shallow breathing index, calculated as RR divided by VT; MV minute ventilation volume; AUROC area under the receiver operating characteristic; CI confidence interval. The cutoff values for indicators were determined by ROC analyses (Youden Index).

# Table S8 Arterial blood gas before and after the SBT.

|  | Weaning Success | Weaning Failure | P value |
| --- | --- | --- | --- |
| Baseline |  |  |  |
| pH | 7.45±0.05 | 7.45±0.04 | 0.54 |
| PaO_2_ | 110±28 | 102±23 | 0.17 |
| PaCO_2_ | 40±6 | 38±4 | 0.45 |
| HCO_3_^-^ | 23±9 | 28±4 | 0.06 |
| BE | 6±9 | 4±4 | 0.5 |
| After the SBT |  |  |  |
| pH | 7.44±0.04 | 7.44±0.04 | 0.89 |
| PaO_2_ | 104±17 | 99±21 | 0.36 |
| PaCO_2_ | 40±4 | 41±7 | 0.40 |
| HCO_3_^-^ | 26±5 | 28±5 | 0.29 |
| BE | 4±7 | 4±4 | 0.95 |

# Table S9 Spearman correlation analysis for Ventilation days before first SBT

|  | Correlation factors | P value |
| --- | --- | --- |
| Diaphragm excursion | -0.36 | **0.04** |
| Diaphragm thicken fraction | -0.01 | 0.96 |
| EAdi | 0.21 | 0.22 |
| EAdi_AUC_ | 0.11 | 0.53 |
| Neuro-ventilatory efficiency | -0.17 | 0.31 |
| Neuro-excursion efficiency | -0.37 | **0.03** |
